# Radiation-induced osteosarcoma of the maxilla and mandible after radiotherapy for nasopharyngeal carcinoma

**DOI:** 10.1186/s40880-016-0153-8

**Published:** 2016-10-12

**Authors:** Lie-Qiang Liao, Hong-Hong Yan, Jun-Hao Mai, Wei-Wei Liu, Hao Li, Zhu-Ming Guo, Zong-Yuan Zeng, Xue-Kui Liu

**Affiliations:** 1 State Key Laboratory of Oncology in South China, Collaborative Innovation Center for Cancer Medicine, Sun Yat-sen University Cancer Center, Guangzhou, 510060 Guangdong P. R. China; 2 Department of Head and Neck Surgery, Sun Yat-sen University Cancer Center, 651 Dongfeng Road East, Guangzhou, 510060 Guangdong P. R. China

**Keywords:** Radiation-induced osteosarcoma, Maxilla and mandible, Nasopharyngeal carcinoma, Prognosis

## Abstract

**Background:**

The increasing incidence of radiation-induced osteosarcoma of the maxilla and mandible (RIOSM) has become a significant problem that can limit long-term survival. The purpose of this study was to analyze the association of clinicopathologic characteristics with treatment outcomes and prognostic factors of patients who developed RIOSM after undergoing radiotherapy for nasopharyngeal carcinoma (NPC).

**Methods:**

We reviewed the medical records of 53,760 NPC patients admitted to Sun Yat-sen University Cancer Center during the period August 1964 to August 2012. Of these patients, 47 who developed RISOM and met inclusion criteria were included in this study. Two of these 47 patients refused treatment and were then excluded.

**Results:**

For all patients treated for NPC at Sun Yat-sen University Cancer Center during the study period, the total incidence of RIOSM after radiotherapy was 0.084% (47/53,760). Two patients (4.4%) had metastases at the diagnosis of RIOSM. Thirty-nine of the 45 (86.7%) patients underwent surgery for RIOSM; most patients (24/39; 61.5%) who underwent resection had gross clear margins, with 15 patients (38.5%) having either a gross or microscopic positive margin. All patients died. The 1-, 2-, and 3-year overall survival (OS) rates for the entire cohort of 45 patients were 53.3%, 35.6% and 13.5%, respectively. The independent prognostic factors associated with high OS rate were tumor size and treatment type.

**Conclusions:**

RISOM after radiotherapy for NPC is aggressive and often eludes early detection and timely intervention. Surgery combined with postoperative chemotherapy might be an effective treatment to improve patient survival.

## Background

Most osteosarcomas arise in normal bone spontaneously, but it has been reported that approximately 5.5% of all osteosarcomas are caused by radiation exposure [1]. Radiation-induced osteosarcoma (RIOS) is a rare and potential late complication of radiotherapy for diseases including Hodgkin’s disease, retinoblastoma, breast cancer, and pelvic cancer.

Nasopharyngeal carcinoma (NPC) has a distinct geographic distribution: the incidence is generally less than 1 per 100,000 person-year in most areas of the world, but the incidence is as high as 20–50 per 100,000 person-years in southern China and southeastern Asia [1–4]. The most effective treatment for NPC is radiotherapy [5]. Unfortunately, radiotherapy can cause serious complications, and the risk of late complications in irradiated tissues must be considered when radiotherapy is prescribed with curative intent. The target volumes in NPC include the skull base, as well as the maxilla, mandible, and pterygoid bone, and RIOS may arise in these sites as a late complication of radiotherapy.

RIOS of the maxilla and mandible (RIOSM) is an uncommon, aggressive tumor that occurs 5 years or more after radiotherapy [1, 5]. Management of RISOM is challenging, and little has been reported in the English-language literature about the characteristics and prognosis of RIOSM after radiotherapy for NPC. The purpose of this study was to analyze the association of clinicopathologic characteristics with treatment outcomes and prognostic factors of patients who developed RIOSM after undergoing radiotherapy for NPC.

### Patients and methods

#### Patient selection

We reviewed the medical records of 53,760 NPC patients admitted to Sun Yat-sen University Cancer Center in Guangzhou, Guangdong, China, between August 1964 and August 2012 and identified 1074 cases of bone sarcoma. Based on the criteria for radiation-induced sarcoma published by Cahan et al. [6], we selected the patients who met the diagnostic guidelines for RIOSM as follows: (1) the neoplasm originated in the irradiated field; (2) the initial bone condition was non-malignant in nature (this criterion was subsequently modified by Arlen et al. [7] to “tumors developed in bone not known to have a primary malignant osteoplastic lesion when the radiotherapy was given”); (3) the neoplasm was histologically diagnosed as osteosarcoma; and (4) there was a relatively long latency period.

#### Follow-up and statistical analysis

The latency period was defined as from the date of the first irradiation treatment of NPC to the date of pathologic diagnosis of RIOSM [6]. Follow-up duration was calculated from the date of diagnosis of RIOSM to the date of last follow-up. The cutoff time was the date of the last contact of these patients or the date of last follow-up. The occurrence rate of RIOSM in patients with NPC treated with radiotherapy was calculated by analyzing the medical records of all patients with NPC treated at Sun Yat-sen University Cancer Center during the study period.

The following variables were analyzed with respect to survival: (1) patient factors: age and sex; (2) treatment of NPC: irradiation course and radiation dose (Gy); (3) RIOSM tumor factors: TNM stage, tumor site, and tumor size; (4) RIOSM pathologic factors: status of surgical margins; and (5) treatment of RIOSM: surgery, radiotherapy, chemotherapy, or a combination of these strategies.

Overall survival (OS) was calculated from the date of RIOSM diagnosis to the date of either death or the last follow-up. OS rates were estimated using the Kaplan–Meier method and compared using the log-rank test. All statistical analyses were performed using SPSS version 22.0 software (SPSS Inc., Chicago, IL, USA). *P* values less than 0.05 were considered statistically significant.

## Results

### Characteristics of patients with RIOSM after radiotherapy for NPC

Forty-seven patients who developed RIOSM after radiotherapy for NPC were eligible for this study; however, two patients who had major complications and refused treatment or were managed conservatively were therefore excluded from the study. For all patients treated for NPC at our hospital during the study period, the total occurrence rate of RIOSM after radiotherapy for NPC was 0.084% (47/53,760). The clinicopathologic characteristics of all 45 included patients (33 men and 12 women) who were diagnosed with RIOSM after radiotherapy for NPC are listed in Table 1. When the patients first presented with NPC, they aged from 13 to 64 years (median 38 years); at diagnosis of RIOSM, they aged from 18 to 69 years (median 49 years). Of the 45 patients with RIOSM, 22 (48.9%) had a tumor ≤5 cm in diameter, and 23 (51.1%) had a tumor >5 cm; 33 (73.3%) arose RIOSM in the maxilla, and 12 (26.7%) in the mandible. Two patients (4.4%) had metastases at diagnosis of RIOSM. Thirty-nine of the 45 patients (86.7%) underwent surgery for RIOSM; of the 39 patients, 24 (61.5%) who underwent resection had gross clear margins, and 15 (38.5%) had either a gross or microscopic positive margin.

**Table 1:** The clinicopathologic characteristics of 45 patients who were diagnosed with radiation-induced osteosarcoma of the maxilla and mandible (RIOSM) after undergoing radiotherapy for nasopharyngeal carcinoma

Characteristic	No. of patients (%)
Sex	
Men	33 (73.3)
Women	12 (26.7)
Age (years)	
≤49	21 (46.7)
>49	24 (53.3)
Tumor size (cm)	
≤5	22 (48.9)
>5	23 (51.1)
Tumor site	
Maxilla	33 (73.3)
Mandible	12 (26.7)
Latency period (years)	
≤8.0	23 (51.1)
>8.0	22 (48.9)
Margin status^a^	
Negative	24 (61.5)
Positive	15 (38.5)

^a^Only 39 of 45 patients underwent surgery

### Latency between radiotherapy for NPC and RIOSM

The median latency period was 8.0 years (range 3.0–34.0 years). RIOSM developed within 5 years after radiotherapy in 6 patients (13.3%); in 13 patients (28.9%), the latency was >10 years. The latency to RIOSM was significantly shorter for patients who received a radiation dose >68 Gy than for those who received ≤68 Gy (median 13.6 vs. 8.0 years, *P* = 0.005). The latency was not significantly different among the groups of patients who received orthovoltage, cobalt-60, or megavoltage X-rays (*P* = 0.569), between men and women (*P* = 0.464), or between patients aged ≤38 and >38 years (*P* = 0.848).

#### Treatment of RIOSM

Of the 45 patients, 6 (13.3%) who either had non-resectable lesions or rejected surgery received chemotherapy alone; 39 (86.7%) underwent surgery, including 30 (66.7%) who underwent surgery only, 8 (17.8%) who underwent surgery and also received chemotherapy, and 1 (2.2%) who received surgery, chemotherapy, and radiotherapy. After extensive resection, two patients underwent reconstruction with a free anterolateral femoral skin flap, and two patients underwent regional flap reconstruction, including a temporalis muscle flap and a pectoralis major myocutaneous flap. All reconstructions were successful. Of the 39 patients undergoing radical excision, 24 (61.5%) achieved gross negative margins, and 15 (38.5%) had gross or microscopic positive margins. Eight patients, including one who underwent complete resection and seven who underwent incomplete resection, received postoperative chemotherapy on an individualized basis. One patient underwent surgery with incomplete resection; this patient received chemotherapy plus radiotherapy (median dose, 60 Gy). For this retrospective study, detailed records of the chemotherapy drugs were not available.

### Follow-up and outcome

**Fig. 1:**
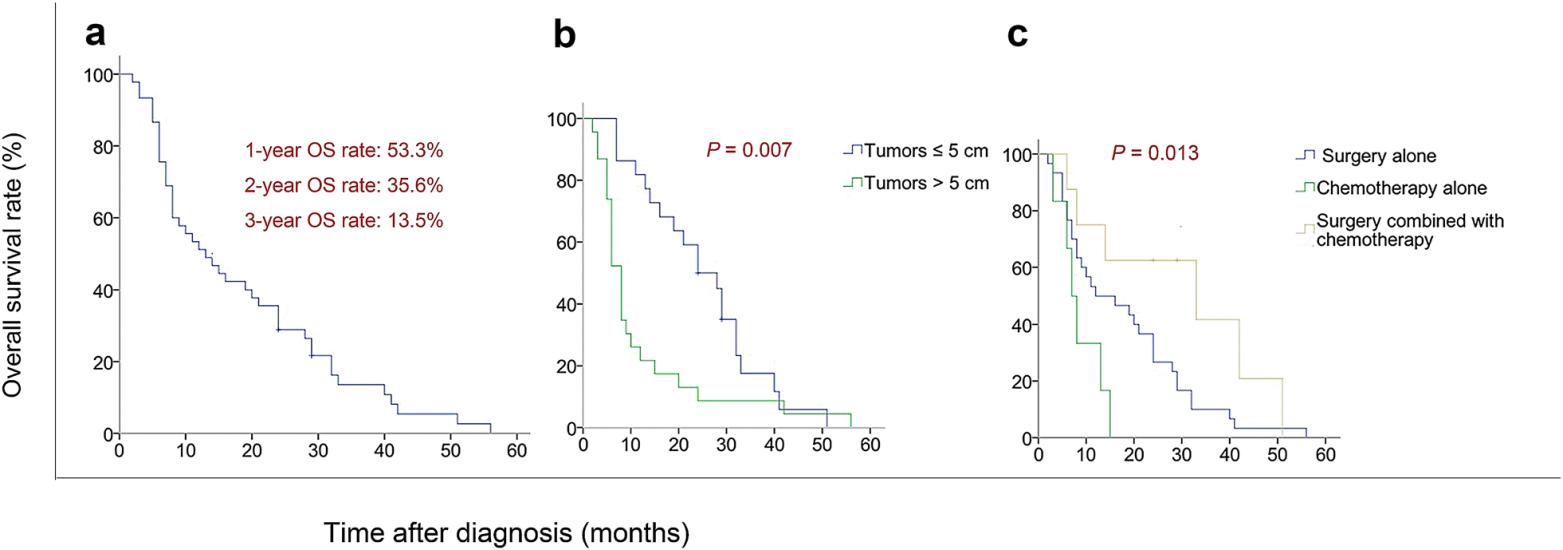
Overall survival (OS) curve for 45 patients with radiation-induced osteosarcoma of the maxilla and mandible (RIOSM). **a** OS curve for 45 patients with RIOSM who underwent radiotherapy for nasopharyngeal carcinoma. **b** OS for two groups stratified by different diameter. **c** OS for three groups stratified by different treatments

For the censored data analysis, the cutoff date for last follow-up was August 31, 2014. The mean follow-up duration was 17.9 months (range 2.1–56.5 months). All patients died before the last follow-up; the 1-, 2-, and 3-year OS rates for the entire cohort of 45 patients were 53.3%, 35.6% and 13.5%, respectively (Fig. 1a). Of the 39 patients who underwent surgery for RIOSM, 10 developed tumor recurrence and all died of disease within 1–10 months after diagnosis of tumor recurrence (median 8.0 months). The interval between surgery and tumor recurrence ranged from 5.0 to 65.5 months (median 16.9 months). Of the patients who experienced tumor recurrence, 6 (60.0%) recurred in the first year, 3 (30.0%) recurred in the second year, and 1 (10.0%) recurred in the third year; 2 (20.0%) had received surgery, 4 (40.0%) had received palliative chemotherapy, and 4 (40.0%) had received conservative therapy. The two patients with RIOSM who were excluded from the study because of treatment refusal both died within 2 months of diagnosis of RIOSM.

### Prognostic factors for OS of patients with RIOSM

The univariate analysis results showed that RIOSM tumor size and treatment type were prognostic factors for OS (Table 2). The 1- and 2-year OS rates were 81.8% and 59.1%, respectively, for patients with tumors ≤5 cm and were 26.1% and 13.0%, respectively, for patients with tumors >5 cm (*P* = 0.007; Fig. 1b). In this cohort, only one patient received a combination of surgery, chemotherapy, and radiotherapy. Therefore, to analyze the effect of treatment, we stratified the remaining 44 patients into three groups: surgery alone (30 patients), chemotherapy alone (6 patients), and combined surgery and chemotherapy (8 patients). Median OS was 14.4 months for the surgery-alone group, 9.0 months for the chemotherapy-alone group, and 33.6 months for the combined surgery and chemotherapy group. The 1- and 2-year actuarial OS rates for the surgery-alone group were 53.3% and 36.7%, respectively, compared with 33.3% and 0.0%, respectively, for the chemotherapy-alone group and 75.0% and 62.5%, respectively, for the combined surgery and chemotherapy group (*P* = 0.013; Fig. 1c). Using the average values as cutoff points, patient age, radiation dose, and latency were not significant prognostic factors for OS. Moreover, the univariate analysis results showed that sex, TMN stage, irradiation source and recurrence were also not significantly associated with OS (Table 2). Surprisingly, in multivariate analysis, margin status was not a significant prognostic factor. We performed additional analysis of OS based on the margin status in the 39 patients who received surgery alone. The 1- and 2-year actuarial OS rates for the 19 patients with negative margins were 63.1% and 47.0%, respectively, versus 40.2% and 20.1%, respectively, for the 10 patients with positive margins (*P* = 0.029 for 1-year OS rates and *P* = 0.023 for 2-year OS rates).

**Table 2:** Association of overall survival with the characteristics of 45 patients with RIOSM

Characteristic	No. of patients	Survival rate (%)		*P*
		1-year	2-year	
Age (years)				
≤49	21	52.4	42.9	0.676
>49	24	54.2	29.2	
Sex				
Women	12	41.7	33.3	0.572
Men	33	57.6	36.4	
Size (cm)				
≤5	22	81.8	59.1	0.007
>5	23	26.1	13.0	
TNM stage				
I/II	19	52.6	36.8	0.159
III/IV	26	53.8	34.6	
Irradiation course^a^				
Split	15	46.7	33.3	0.721
Continuous	27	55.6	37.0	
Radiation dose (Gy)				
≤68	22	59.1	45.5	0.129
>68	23	47.8	26.1	
Latency period (years)				
≤8.0	23	60.9	39.1	0.699
>8.0	22	40.9	31.8	

^a^Three patients did not have available information

## Discussion

Although radiotherapy is an effective treatment of NPC, it also has the potential to cause secondary malignancies. The incidence of RIOSM is on the rise due to improved early discovery and treatments of NPC [1–3]. In the English-language literature, one study reported a cumulative occurrence rate of 0.03%–0.8% of RIOSM after radiotherapy in patients with NPC [2]. Liu et al. [1] estimated that the occurrence rate of RIOSM in patients with NPC was nearly 0.037%; of the 15 cases they studied, five tumors arose in the maxilla, seven in the mandible, and three in the junction of the nasal cavity and para-nasal sinuses. In our study, we considered RIOSM arising in the maxilla (33 cases) and mandible (12 cases) only, with a maxilla-versus-mandible ratio of 2.75:1, corresponding to an estimated cumulative occurrence rate of 0.084%.

Radiotherapy is the most common treatment of NPC, and the irradiation fields may extend from the skull base down to the lower neck region. As a late complication of radiotherapy, osteosarcoma can arise in the maxilla and mandible. Some researchers have suggested that RIOSM is most likely to occur after exposure to doses <30 Gy [6, 7]. In our study, the mean dose that patients received was 68 Gy, which was much higher than the mean dose of 45 Gy (range 25–110 Gy) that was reported in a previous study of RIOS [8]. Since the maxilla and mandible were not as routine conservation tissue it is difficult to calculate the exact radiation dose on the bones in NPC radiotherapy. Moreover, many uncertainties can affect the calculation of radiation dose, such as scatter dose; a previous study pointed out that a high scatter dose would cause scattering low dose ratio, which induced malignancies [9]. As radiotherapy technology advances, ionizing radiation dose may affect the occurrence rate of RIOSM. However, we could not obtain exact data on the radiation dose that would increase the occurrence rate of secondary malignancies.

Intensity-modulated radiotherapy (IMRT) has enabled target volumes to be more precisely, but IMRT actually increases the volume of normal tissue subjected to low-dose ionizing radiation [10]. Some researchers have suggested that medium-to-low-dose radiation may induce carcinogenesis more effectively than high-dose radiation [5, 10]. Moreover, some studies have found that concurrent chemotherapy significantly improved treatment outcomes in NPC patients, although others have suggested that chemotherapy may increase the risk of radiation-induced sarcoma [11, 12]. We made two speculations: first, whether IMRT indeed increases the occurrence rate of secondary malignancies; and second, whether IMRT did not increase the risk of secondary cancer. These speculations should be tested in future studies with a longer follow-up and a larger number of patients by analyzing clinicopathologic data. As more patients with NPC are treated with irradiation and now survive longer, the incidence of RIOSM is likely to increase [13, 14].

Previous studies reported latencies ranging from 5.0 to 30.0 years (mean 12.9 years) [15]. Similarly, in the present retrospective study, the median latency of RIOSM was 8.0 years (range 3.0–34.0 years). The factors that influence latency are unknown. In this cohort, patients who received radiation doses >68 Gy had a significantly shorter latency than patients who received ≤68 Gy (*P* = 0.005). However, the radiation source (orthovoltage, cobalt-60, or megavoltage X-rays) and patient sex or age had no significant effect on latency.

RIOSM is an aggressive tumor with a very poor prognosis. Most studies have reported low 5-year OS rates, ranging from 10% to 30% [16–21]. In the most recent study reported by Tabone et al. [22], the 8-year OS and disease-free survival rates for 23 patients with RIOSM were 50% and 41%, respectively, suggesting that the prognosis of patients with RIOSM has improved over time. However, in our cohort, the 1-, 2-, and 3-year actuarial OS rates for patients with RIOSM were 53.3%, 35.6% and 13.5%, respectively. Moreover, all patients died within 5 years after RIOSM diagnosis. Other studies have reported that the 5-year OS rate of patients with primary maxillofacial osteosarcoma was 44%–70% [6, 7, 13, 23, 24]. RISOM results in worse outcomes compared with stage-matched osteogenic sarcomas of the jaw. Thiagarajan et al. [25] suggested that the poor outcomes were due to the following reasons: (1) delayed diagnosis in previously irradiated tissue; (2) compromised resection margins, due to proximity of the tumor to critical structures; (3) limited treatment options in a maximally irradiated field (i.e., technical difficulties of operating within an irradiated field and difficulties with irradiation to the field with surrounding normal tissues, which have been treated to near tolerance); (4) poor tumor sensitivity to chemotherapy; (5) the high-grade nature of the vast majority of RIOS; and (6) host immunosuppression caused by a combination of tumor-related factors and previous treatment [1, 26, 27]. This study clearly indicates that the prognosis of patients with RIOS in the maxilla and mandible is poorer than patients with primary osteosarcoma of the maxilla and mandible.

The incidence of radiation-induced sarcoma of head and neck (RISHN) is increasing, with an estimated risk of up to 0.3% [2, 23]. RISHN development may be influenced by radiation dose, age at initial exposure, exposure to chemotherapeutic agents, and genetic features. RISHN is associated with poor outcomes, and surgical resection with clear margins seems to offer the best chance for cure [23]. Management of RISOM is more challenging, entailing surgery for irradiated tissue and a limited scope for further radiotherapy and chemotherapy.

Treatment for RIOSM includes surgery, radiotherapy, chemotherapy, or a combination of these strategies. Complete surgical excision seems to be necessary for the treatment of radiation-induced sarcoma; however, in patients with RIOSM, radical surgery is suitable only for early-stage tumors [1]. Although preoperative assessment can help determine the surgical boundaries, it is difficult for surgeons to judge whether the tumor has already invaded to the surrounding area. Moreover, RIOSM often occurs close to important structures such as the carotid artery and skull base; radical surgery in these regions is associated with a high risk of critical damage to important structures. The significance of chemotherapy for RIOSM is under debate: some researchers have concluded that RIOSM is insensitive to chemotherapy, whereas others have stated that chemotherapy is effective [1, 25, 28]. One study reported that a combination of surgery and chemotherapy resulted in a higher OS rate than either surgery alone or chemotherapy alone [29]. In our cohort, 39 patients underwent surgery; of these 39 patients, 15 (38.5%) had gross or microscopic positive margins. In multivariate analysis, margin status was not significantly associated with OS. However, for patients who received surgery alone, a negative margin was associated with significantly higher 1- and 2-year OS rates (19 patients; 63.1% and 47.0%, respectively) than a positive margin (10 patients; 40.2% and 20.1%, respectively; *P* = 0.029, *P* = 0.018). We suggest that radical surgery with a negative margin leads to a significantly better prognosis for patients with RIOSM. In our cohort, chemotherapy alone did not influence survival. Therefore, surgery combined with postoperative chemotherapy may be an effective strategy to improve survival for patients with RIOSM. In our cohort, since only one patient received combined surgery, chemotherapy, and radiotherapy, we cannot make any definitive conclusions about the effectiveness of combined treatment for RIOSM. In fact, it has been reported that radiotherapy provides no survival benefit for patients with RIOSM [18, 21, 30–32].

In our cohort, RIOSM tumor size had prognostic significance. In agreement with the results of other studies [3, 23–27], we found that a larger tumor was more likely to be associated with more advanced disease and resulted in a poorer treatment outcome. Furthermore, in cases with large tumors surrounded by vital tissues, it is extremely risky and difficult to perform radical surgery and achieve a negative margin.

Unlike other reports [7, 21, 33], in our study recurrence was not prognostic for disease control status. RIOSM is an aggressive sarcoma, and, in our study, the mean OS time was 14.2 months, and only 20 patients survived for more than 16 months. However, the interval between surgery and tumor recurrence ranged from 5.0 to 65.5 months (mean 16.9 months). We contend that all of the deaths in our study were due to RIOSM. However, only a small proportion of RIOSM cases in our study could be detected on clinical examination and received appropriate treatment.

The potential limitations of our study are its retrospective nature, the relatively small sample size, and the fact that it was performed at a single institution. Given the rarity of this complication, larger multi-center prospective studies should be conducted to confirm these preliminary results and further analyze the treatment outcomes.

## Conclusions

Our study confirmed the low occurrence rate and poor prognosis of RIOSM in patients with NPC. Complete surgical resection was a significant prognostic factor for survival. Surgery combined with postoperative chemotherapy may be an effective strategy to improve survival for patients with RIOSM. We concluded that long-term follow-up is necessary for the early detection of RIOSM in patients with NPC.
